# *OPERAnet*, a multimodal activity recognition dataset acquired from radio frequency and vision-based sensors

**DOI:** 10.1038/s41597-022-01573-2

**Published:** 2022-08-03

**Authors:** Mohammud J. Bocus, Wenda Li, Shelly Vishwakarma, Roget Kou, Chong Tang, Karl Woodbridge, Ian Craddock, Ryan McConville, Raul Santos-Rodriguez, Kevin Chetty, Robert Piechocki

**Affiliations:** 1 School of Computer Science, Electrical and Electronic Engineering, and Engineering Maths, University of Bristol, Bristol, BS8 1UB UK; 2 Department of Security and Crime Science, University College London, London, WC1H 9EZ UK

**Keywords:** Computer science, Electrical and electronic engineering

## Abstract

This paper presents a comprehensive dataset intended to evaluate passive Human Activity Recognition (HAR) and localization techniques with measurements obtained from synchronized Radio-Frequency (RF) devices and vision-based sensors. The dataset consists of RF data including Channel State Information (CSI) extracted from a WiFi Network Interface Card (NIC), Passive WiFi Radar (PWR) built upon a Software Defined Radio (SDR) platform, and Ultra-Wideband (UWB) signals acquired via commercial off-the-shelf hardware. It also consists of vision/Infra-red based data acquired from Kinect sensors. Approximately 8 hours of annotated measurements are provided, which are collected across two rooms from 6 participants performing 6 daily activities. This dataset can be exploited to advance WiFi and vision-based HAR, for example, using pattern recognition, skeletal representation, deep learning algorithms or other novel approaches to accurately recognize human activities. Furthermore, it can potentially be used to passively track a human in an indoor environment. Such datasets are key tools required for the development of new algorithms and methods in the context of smart homes, elderly care, and surveillance applications.

## Abstract (summary)

**Table:** 

Measurement(s)	Human physical activity • Human location
Technology Type(s)	WiFi sensing device • ultra-wideband impulse radar • passive WiFi radar • Kinect motion sensor
Factor Type(s)	Human location • Human physical activity • Room geometry • Participant demographics • Contactless sensing devices
Sample Characteristic - Organism	Homo sapiens
Sample Characteristic - Environment	Office building
Sample Characteristic - Location	United Kingdom

## Background & Summary

Over the past few years, Internet of Things (IoT) has become a key enabler for various applications since it supports the exchange of ubiquitous data or information among smart devices or sensors with little to no human intervention^1^. It has revolutionized research in Human Activity Recognition (HAR) techniques due to their potential applications in areas such as healthcare (e.g., monitoring elderly people or those with disabilities), smart homes (e.g., controlling appliances based on human activities for achieving efficient energy consumption), surveillance and security, virtual gaming, among others. Numerous techniques have been proposed for HAR, ranging from inertial/wearable sensors^2–6^, vision-based methods such as Microsoft Xbox Kinect sensor^7^, to unobtrusive methods based on Radio-Frequency (RF) waves such as WiFi Channel State Information (CSI)^8^, Passive WiFi Radar (PWR)^9^ and Ultra-Wideband (UWB)^10^. Recognizing human activities, especially using RF signals, is a challenge and open-source datasets would help in devising techniques and algorithms that can boost research in this field, which can ultimately lead to standardization. To date many datasets acquired from RF, vision and inertial sensors have been published, which are intended for a number of applications. These include WiFi CSI-based activity recognition^11–15^, sign language recognition^16^, fall detection^17^, device-to-device localization^18^, or UWB-based gesture recognition^19^, motion detection/recognition^20–22^, passive target localization^23^, people counting^24^, or active radar-based sensing^25–28^, as well as physical activity recognition using inertial/wearable sensors^29–34^, while others have proposed action recognition datasets acquired from vision and motion capture systems^35–38^. However, most of these databases have some shortcomings in the layout and number of sensors, which cannot fully represent the human activity features. First, most of these datasets comprise of measurements from a single sensor. Second, the datasets are limited by the number of human activities being captured and the layout used for capturing the datasets. Compared with unimodal sensors, multimodal sensors can collect different types of data which differ in characteristics, including dimensionality, distribution, and sparsity. Fusion of multimodal data can help in describing human actions more accurately. As compared to the aforementioned works and to the best of the authors’ knowledge, this is the first work to propose a multimodal dataset comprising of RF and vision-based methods that is intended not only for the sensing of day-to-day activities but also for passive (uncooperative) localization. The contributions herein are:

- Multimodal data collection intended for human activity recognition and passive localization, i.e, the targets are oblivious to these processes (non-collaborative) and they only reflect or scatter the signals from the transmitter to receivers. Most datasets consider only one particular modality such as either UWB, WiFi CSI, PWR or Kinect, independently. In this work, we consider multiple synchronized modalities. Experiments span across two environments which can be used for investigating sensing in complex or untrained environments.
- Approximately 8 hours of measurements are fully annotated with location and activity labels of high temporal resolution, capturing the participant’s movement and natural behaviour within the monitoring area, as would be the case in a real-world environment. The dataset is comprehensive in so far it contains over 1 Million annotated data points.
- The presented data can be exploited to advance human activity recognition technology in different ways, for example, using various pattern recognition and deep learning algorithms to accurately recognize human activities. For this purpose, the users can apply different signal processing pipelines to analyze the recorded WiFi CSI, PWR, UWB and Kinect data and extract salient features that can be used to recognize the human activities and/or concurrently track the target’s position within an indoor environment.
- This is the first dataset that is collected with an explicit aim to accelerate the development of self-supervised learning techniques. Such techniques are extremely data hungry, requiring orders of magnitude larger datasets compared to more traditional supervised learning. Furthermore, each modality consists of multiple receivers and this corresponds to multiple views of the data which can undoubtedly be used in multimodal and multiview data fusion networks for improving the performance in concurrent activity recognition and localization tasks.

This open-source dataset is intended for both HAR and non-cooperative localization, which are areas of growing interest to research communities including but not limited to radar, wireless sensing, IoT and computer vision. To ensure that the dataset aligns to the FAIR (Findable, Accessible, Interoperable, Reusable) Data principles of Open Science, we have (i) made it publicly available for download via the Figshare portal, (ii) provided an in-depth and clear description of the dataset for each modality, (iii) formatted our dataset using standard filetypes and encoding, and (iv) provided example scripts/codes that will allow the user to load and analyze the data from each modality.

## Methods

**Fig. 1:**
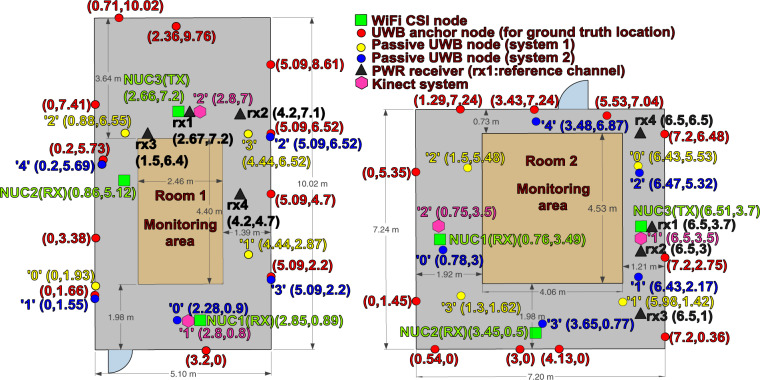
Experiment rooms layouts.

Experiments were performed in a university environment in two furnished rooms, with desks, chairs, screens, and other office objects lying in the surroundings. The room layouts are depicted in Fig. 1 along with their physical dimensions. A maximum of six subjects of different age groups participated in the experiments which were intended for the sensing of day-to-day activities as well as non-collaborative localization.

Our dataset^39^ is composed of approximately 8 hours of measurements that were collected across multiple modalities including WiFi Channel State Information (CSI), Ultra-Wideband (UWB), Passive WiFi Radar (PWR) and Kinect sensor systems. The breakdown of the activities’ durations is given in Table 1. The monitoring devices were installed on the extremity (boundary) of the rooms such that enclosed spaces of dimensions 2.46 m × 4.40 m and 4.06 m × 4.53 m were used as monitoring areas for Room 1 and 2, respectively. The description of the various experiments performed is provided in Table 2.

**Table 1:** Breakdown of experiment activities in terms of duration (minutes).

Activity	Duration (minutes)
Background	18.3465
Sit on chair	35.2455
Stand from chair	34.8754
Walk	75.9323
Lie down	26.6891
Stand from the floor	26.4151
Upper body rotate	74.5893
Steady state (no activity)	124.8624
Crowd counting	27.4127
Localization (CSI receiver NUC2 only)	18.1803

Even though no personal data has been collected from the participants during the experiments, each participant was still fully informed about the purpose of the study and what was expected of them. Informed consent was obtained from each participant prior to the experiments. All studies that fall under the OPERA - Opportunistic Passive Radar for Non-Cooperative Contextual Sensing project were thoroughly reviewed and fully approved by the University of Bristol Faculty of Engineering Research Ethics Committee (application number: 96648). Risk assessment was also carried out and approved prior to the experiments.

**Fig. 2:**
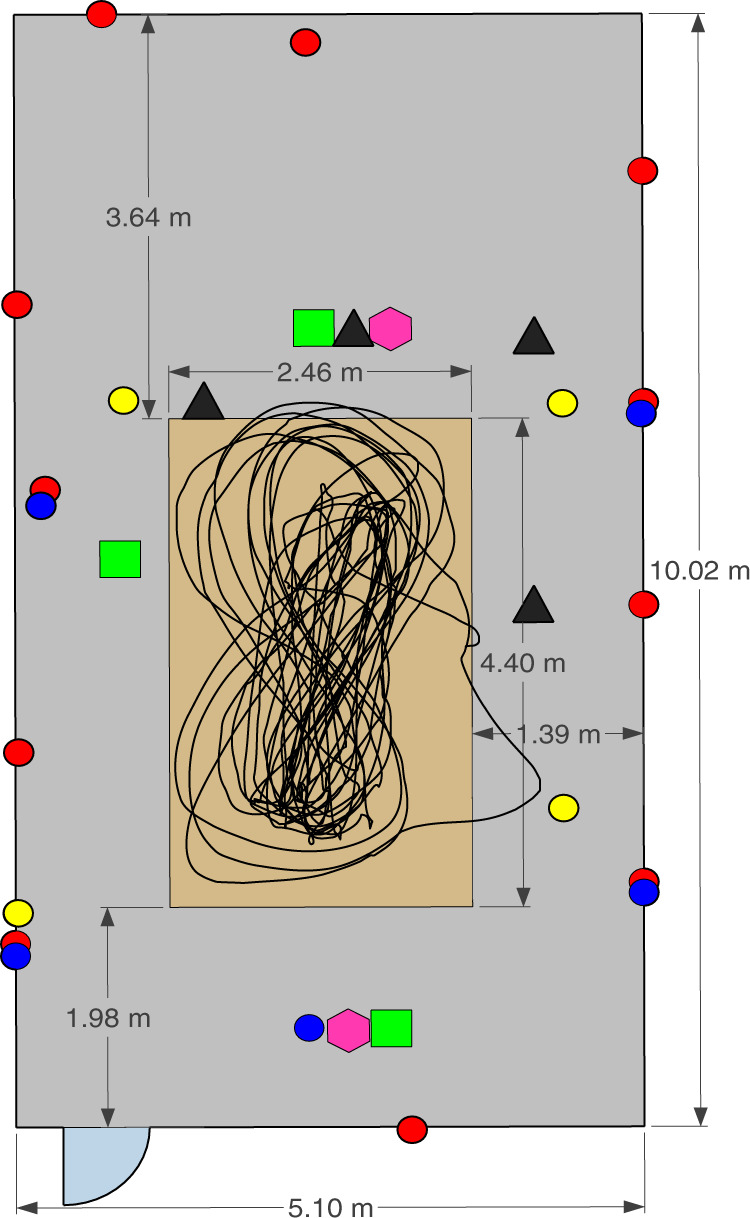
Crowd counting experiment (exp028) in Room 1. This picture shows the last person’s walking path after the 5 previous participants have stepped out of the room sequentially.

**Fig. 3:**
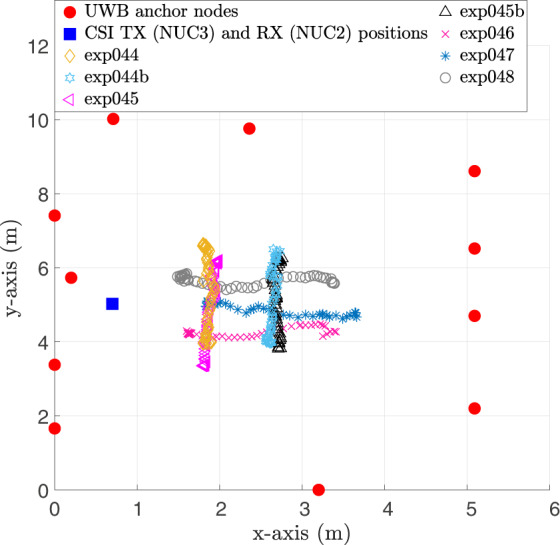
Figure showing paths walked by target in dynamic CSI localization experiments (exp044–exp048).

**Fig. 4:**
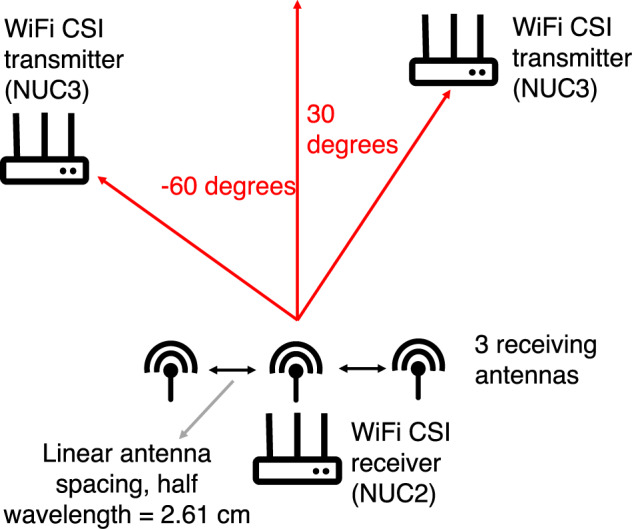
Device-to-device CSI localization experiment setup (exp049-exp054).

Referring to the experiment numbers in Table 2, exp001–exp054 were performed in Room 1 while exp055–exp061 were carried out in Room 2. exp028 is the crowd counting experiment whereby six people walked randomly and continuously within the monitoring area of Room 1. Then, after approximately every 5 minutes, one person moved out of the room. Figure 2 shows the particular instant of exp028 where 5 out of the 6 people already left the monitoring area and only the last person’s ground truth walking trajectory is shown. For illustration purposes only, a moving average filter is applied to the raw ground truth positions to smooth the target’s trajectory path. The experiments exp034–exp048 (exp034 is the empty room background data) were device-free localization experiments involving a human target who was standing still at several positions or walking along a straight short path in a number of directions as shown in Fig. 3. The target wore a tag to get his/her ground truth position. Note that only the WiFi CSI transmitter (NUC3) and receiver (NUC2) were used during these experiments for recording data and they were placed side by side. As for the device-to-device localization experiments (exp049–exp054), the CSI transmitter (NUC3) and receiver (NUC2) were placed at different angles with respect to each other (0°, 30°, −30°, 60°, −60°), as shown in Fig. 4 (no human target). Note that one tag was placed on the CSI transmitter and another on the receiver to get their fixed ground truth locations within the environment.

**Table 2:** Experiment description.

Experiment no.	Details
exp001, exp019, exp034, exp055	Background (1) data (empty room).
exp002, exp006, exp010, exp014, exp020, exp024	Person walking (2).
exp003, exp007, exp011, exp015, exp021, exp025	Person sitting (3) and standing from chair (4).
exp004, exp008, exp012, exp016, exp022, exp026	Person lying down on floor (5) and standing up from floor (6).
exp005, exp009, exp013, exp017, exp023, exp027	Person rotating upper-half of his/her body (7).
exp018, exp029-exp033	Person performing the six activities (2–7) continuously and randomly (no predefined order).
exp056-exp061	Person performing the six activities (2–7) in a predefined order, starting with activity “walking” and ending with activity “body rotating”.
exp028	Crowd counting. A maximum of six people walking continuously and randomly. Experiment starts with six people and then after every 5 minutes, one person steps out of the monitoring area.
exp035-exp043	Device-free static localization. CSI transmitter (NUC3) and CSI receiver (NUC2) are placed side by side and the target stand still at a given position for each experiment number.
exp044-exp048	Device-free dynamic localization. CSI transmitter (NUC3) and CSI receiver (NUC2) are placed side by side and the target moves along a short straight path for each experiment number.
exp049-exp054	Device-to-device localization. No human target present. CSI transmitter (NUC3) and CSI receiver (NUC2) are placed at different angles with respect to each other (−30°, 0°, +30°, −60°, 0°, +60°) for each experiment number.

### WiFi CSI

The WiFi CSI system consisted of three Intel Core i5 vPro Next Unit of Computing (NUC) devices. Each device was fitted with an Intel 5300 Network Interface Card (NIC). In order to extract the CSI from the NICs, the Linux 802.11n CSI tool^40^ needs to be installed on the devices running an appropriate kernel version of the Linux operating system. Appropriate firmware and drivers need to be installed on the devices in order to expose the CSI. More information regarding the installation steps can be found in^41^. The CSI provides information about the wireless channel characteristics such as multipath propagation, attenuation, phase shift, etc. It is regarded as a fine-grained information since it describes the amplitude and phase information of the signal across multiple overlapping but orthogonal subcarriers in the Orthogonal Frequency Division Multiplexing (OFDM) physical layer waveform. In a WiFi system based on OFDM, the CSI is used by the equalizer in the receiver to reverse the detrimental effects of the channel and recover the transmitted signal. The channel estimate (i.e., CSI) is obtained by transmitting a training sequence (pilot symbols) which is known by both the WiFi transmitter and receiver. This process is often referred to as channel sounding. CSI or Channel Frequency Response (CFR) are often used interchangeably and they represent the wireless channel in the frequency domain. Applying the Inverse Fast Fourier Transform (IFFT) to CFR gives rise to the Channel Impulse Response (CIR) in the time domain and this characterizes the amplitude and phase information over multiple propagation paths. The Intel 5300 NIC extracts CSI over 30 subcarriers, spread evenly among the 56 subcarriers of a 20 MHz WiFi channel or the 114 subcarriers in a 40 MHz channel^40^. To record the CSI, the injector mode was used whereby one NUC was configured as the transmitter (injector) while the receivers were monitoring the channel into which packets were injected. This method requires that both the transmitter and receiver be equipped with the Intel 5300 NIC. In the access point (AP) method, only the receiver needs to be equipped with the Intel 5300 NIC and CSI data is logged at the receiver by pinging an access point. However, this method is not very stable since there might be a lot of dropped packets (e.g., due to interference) and also the packet rate is limited. The CSI data was stored on the receivers, NUC1 and NUC2, for offline processing. The raw data is in .dat format and needs to be parsed by appropriate Matlab/Python utilities^41^ to convert it to a format that can be easily interpreted. Since the Intel 5300 supports Multiple Input Multiple Output (MIMO) capability, the CSI data was logged as a 3D tensor of complex numbers for each received packet, with *n*_*t*_ × *n*_*r*_ × *N*_*sc*_ complex values, where *n*_*t*_ is the number of transmit antennas, *n*_*r*_ is the number of receive antennas and *N*_*sc*_ is the number of subcarriers. The parameters of the CSI system are summarized in Table 3. Referring to the green boxes in Fig. 1, the WiFi CSI system comprised of a single transmitter (NUC3) and two receivers (NUC1 and NUC2). NUC1 was installed facing the transmitter in a Line-of-Sight (LoS) geometry, while receiver NUC2 was placed in a bi-static configuration (90°) with respect to the transmitter.

**Table 3:** WiFi CSI system parameters.

Parameter	Value
WiFi band	5 GHz (channel 149)
NIC	Intel 5300
Subcarriers, *N*_*sc*_	30
Antenna	omni-directional (6 dBi)
Packet rate	1600 Hz
No. of transmit antennas, *n*_*t*_	3
No. of receive antennas, *n*_*r*_	3

### UWB

Three UWB systems were used during the experiments. The first system (see red nodes in Fig. 1) was used to obtain the ground truth position of the target while he/she wore one or more tags and moved within the monitoring area. The update rate, that is, the rate at which the 2D *xy* coordinates were logged for each individual tag was 10 Hz (using Decawave’s DRTLS android app). The other two passive UWB systems (yellow and blue nodes) consisted of fixed nodes installed in a multi-static configuration and which were exchanging CIR data among themselves. UWB system 1 (yellow nodes in Fig. 1) was implemented using four Decawave’s EVK1000^42^ modules. The modules were programmed with a custom firmware so as to record CIR data on all of them. Node ‘0’ was acting as an initiator whereby it exchanged Single-Sided Two-Way Ranging (SS-TWR) messages (*poll*, *response* and *final*) with each of the other 3 nodes. When a given node replies back, the frame is broadcast and heard by all other nodes operating on the same channel. In this way, each node can read the received frames in their accumulator and extract the CIR data. Therefore, CIR data is available in a bidirectional mode between all pairs of nodes. This means that all nodes act as transmitters and receivers, giving rise to a maximum of 12 communication links. The 4 nodes were connected to laptops in order to record the CIR data via a serial terminal.

UWB system 2 (blue nodes in Fig. 1) was implemented using five Decawave’s MDEK1001^43^ modules. These units were also flashed with custom firmware so as to record CIR data on all of them. Node ‘0’ was acting as an initiator and transmitted a packet every 10 ms. The packet essentially includes a time schedule for transmission for the other 4 nodes. In this way, each node knows who needs to transmit next and when with minimal delay. Thus the transmission was performed in a round-robin fashion to avoid collision. Nodes with IDs 1–4 were connected to laptops to record the CIR data via a serial terminal. The average packet rate for UWB system 1 (yellow nodes) was around 400 Hz while for UWB system 2 (blue nodes), the average packet rate was around 195 Hz, considering combined communication links. The other parameters for the three UWB systems are summarized in Table 4.

**Table 4:** UWB systems’ parameters (*maximum receiver bandwidth is approximately 900 MHz).

UWB parameter	Ground truth system (red)	System 1 (yellow)	System 2 (blue)
Channel number	5	4	3
Carrier frequency	6489.6 MHz	3993.6 MHz	4492.8 MHz
Bandwith	499.2 MHz	1331.2* MHz	499.2 MHz
Pulse repetition frequency	64 MHz	64 MHz	64 MHz
Data rate	6.8 Mbps	6.8 Mbps	6.8 Mbps
Preamble length	128 symbols	128 symbols	128 symbols
Preamble acquisition chunk size	8	8	8
Preamble code	9	17	9

The Decawave’s UWB chipset stores the CIR in an accumulator and each tap of the accumulator represents a sampling interval of Δ*τ*_*s*_≈ 1.0016 ns (i.e., half a period of the 499.2 MHz fundamental frequency)^44^. The accumulator spans over one symbol time. This represents 992 and 1016 samples for the nominal Pulse Repetition Frequency (PRF) of 16 MHz and 64 MHz, respectively. Each measured CIR sample is a complex number which can be broken down into its real and imaginary components. Only 35 and 50 CIR samples out of 1016 are considered in the experiments for UWB systems 1 and 2, respectively. These correspond to a sensing range of 10.5 m and 15 m for UWB systems 1 and 2, respectively. Each CIR measurement was read from the accumulator memory starting 3 samples (i.e., 3 ns) before the detected first path index (reported by the FP_INDEX field in register 0x15 of DW1000 chipset) by the Leading Edge Detection (LDE) algorithm. As the CIR magnitudes are dependent on the number of preamble symbols used for CIR accumulation, for each CIR measurement in the UWB datasets, the magnitude values have been normalized using the Preamble Accumulation Count (PAC) value^45^ (see rx_pream_count column in UWB datasets), as reported by the RXPACC register in the DW1000 chipset.

### PWR

For the PWR system, a USRP-2945^46^ was used as the receiver which is equipped with four synchronized channels. The USRP-2945 features a two-stage superheterodyne architecture with four independent receiving channels and shares local oscillators for phase-coherent operation. Each receiving channel was equipped with a 6-dB directional antenna. The collected raw data is then routed to a computing unit through a PCIe port, which is a desktop computer in this work. A PWR system consists of a minimum of two synchronized channels; a *surveillance channel* which records reflected WiFi signals from the monitoring area and a *reference channel* which records the direct signal emitted from the transmitter. As mentioned previously, four channels are used in the USRP-2945, where one channel was used as the reference channel (denoted as “rx1” in Fig. 1) while the other three channels were used as surveillance channels (denoted as “rx2”, “rx3” and “rx4” in Fig. 1). Since the PWR system does not transmit a signal (it only monitors received signals), it can use any third-party signal source as the illuminator but however, a reference signal is needed. In this work, we used the CSI transmitter (NUC3) as the PWR source for convenience, allowing a direct comparison between the two systems’ performance.

PWR correlates the signal from the surveillance and reference channels to estimate two parameters: relative range and Doppler frequency shift. Additionally, a CLEAN^47^ algorithm has been used to remove the direct signal interference. More details on this signal processing can be found in^47^. However, due to the limitation of the WiFi signal bandwidth (40 MHz in this work), the range resolution is limited to 3.75 meters which is too coarse for indoor applications. Therefore, only the Doppler frequency shifts are recorded in the form of Doppler spectrograms. The output from the PWR system is specified as *n*_*s*_ × *n*_*b*_ × *N*_*t*_ real values, where *n*_*s*_ is the number of surveillance channels, *n*_*b*_ is the number of Doppler bins and *N*_*t*_ is the number of time frames. Other details about the PWR’s parameters are given in Table 5. Note that the PWR system recorded the spectrogram data at a measurement rate of 10 Hz. The measurement rate refers to the number of system outputs per second^48^. For the WiFi CSI system, the measurement rate is equal to the number of packets received per second, which is equal to 1.6 kHz. As for the PWR system, the measurement rate is limited by the number of baseband signals that can be processed by the computing device. Therefore, the measurement rate of the PWR system was empirically chosen and set at 10 Hz.

**Table 5:** PWR system parameters.

Parameter	Value
WiFi Band	5 GHz (channel 149) - CSI transmitter
RF frontend	USRP 2945
Antenna	omni-directional (6 dBi)
Packet rate	1600 Hz
Measurement rate	10 Hz
No. of surveillance channels, *n*_*s*_	3
No. of reference channels, *n*_ref_	1
No. of Doppler bins, *n*_*b*_	200

### Kinect

We used two of Microsoft’s Kinect v2 sensors to gather motion capture data from different human activities. Kinect v2 incorporates an infrared depth sensor, a RGB camera, and a four-element microphone array that provides functionalities such as three-dimensional skeletal tracking, facial recognition, and voice recognition. Although the device was originally developed to play games, numerous researchers have used it for applications beyond its initial intended purpose. Due to the low cost and wide availability, it has now been used extensively in research areas such as video surveillance systems where multiple Kinect devices are synchronized to track groups of people even in complete darkness^49^, improve live three-dimensional videoconferencing^50^ and in medical applications to measure a range of conditions such as autism, attention-deficit disorder and obsessive-compulsive disorder in children^51^. Note that in skeleton tracking, Kinect might suffer from occlusion when some parts of the human body are occluded with others and therefore cannot be tracked accurately.

Therefore, in this work, we used two Kinects to track three-dimensional time-varying skeletal information of the human body, including 25 joints such as head center location, knee joints, elbow joints, and shoulder joints from two different directions. The real advantage of using motion capture technology is capturing more accurate, more realistic, and complex human motions. This three-dimensional joint information can further be used for simulating the corresponding radar scatterings mimicking a typical PWR sensing system. In one of our previous works, we presented an open-source motion capture data-driven simulation tool, *SimHumalator*, that can generate large volumes of human micro-Doppler radar data at multiple IEEE WiFi standards (IEEE 802.11g, ax, and ad)^52^. Radar scatterings were simulated by integrating the animation data of humans with IEEE 802.11 compliant WiFi transmissions to capture features that incorporate the diversity of human motion characteristics and the sensor parameters. More importantly, we have demonstrated that the human micro-Doppler data generated using the simulator can be used to augment limited experimental data^53,54^. Interested researchers can download the simulator from https://uwsl.co.uk/. The output from the Kinect system is specified as *N*_*t*_ × *N*_*b*_ × *N*_*d*_ real values, where *N*_*t*_ is the number of time frames, *N*_*b*_ is the number of tracked joints on the human body, and *N*_*d*_ is the three-dimensional position (*x, y, z*) information.

### Ground truthing

- The Decawave (now acquired by Qorvo) MDEK1001 development kit^43^ was used for obtaining the ground truth position of the targets. 11 units were configured as anchors and mounted on walls in the experiment rooms (see red nodes in Fig. 1). Their *xy* coordinates were manually measured using a laser measuring device, which were then entered in the DRTLS Android app. A maximum of 6 tags were configured for exp028, while for the activity recognition experiments, the person wore two tags, one on each arm. Two UWB units were also configured as listeners so as to record the *xy* coordinates of the tags using a serial terminal on two laptops, along with their timestamps with millisecond precision. The MDEK1001 kit features a location update rate of up to 10 Hz for each individual tag. Since two listeners were deployed, this means we can have up to 20 Hz update rate for each individual tag.
- As for the labelling of activities, a program was developed in Matlab with automated voice output to instruct the person when to perform the various activities such as sitting, standing, etc. At the same time, the programmable script recorded the timestamps (with millisecond precision) at which the activity was instructed to be performed. The person just had to listen to the voice command and perform the activity accordingly. As a backup solution, another activity labelling application was developed in Matlab where one can insert the labels for the required activities. Then, an observer constantly looked at the person doing the activities and clicked on the appropriate button in the app to record the start and stop times of the activity. All labels were stored in text files along with their timestamps (with millisecond precision). Note that all modalities were synchronized to the same local Network Time Protocol (NTP) server, resulting in synchronization accuracy across all modalities of <20 ms.

## Data Records

Measurements have been collected across four modalities during the experiments, namely, WiFi CSI, UWB, PWR and Kinect. The dataset can be accessed and downloaded from our Figshare repository^39^. The dataset has been compressed (zipped) into separate folders for each modality, allowing the user to only download the data of interest. The zipped folders’ names and the number of files in each folder, along with their file formats, are specified in Table 6. The directories wificsi1 and wificsi2 refer to the data collected by the WiFi CSI receivers, denoted by “NUC1” and “NUC2” in Fig. 1, respectively. uwb1 and uwb2 refer to the data collected by the two passive UWB systems, represented by the yellow and blue nodes in Fig. 1, respectively. The directory pwr contains the PWR spectrogram data recorded from the three surveillance channels (“rx2”, rx3” and “rx4” represented as black triangles in Fig. 1) for each experiment (excluding exp001, exp019, exp034–exp054). Finally, the directory kinect contains the Kinect sensor data files for each experiment (excluding exp001, exp019, exp028, exp034–exp055).

**Table 6:** Dataset directory details.

Directory name	No. of files	File format
wificsi1	40	.mat
wificsi2	63	.mat
uwb1	40	.csv
uwb2	40	.csv
pwr	38	.mat
kinect	36	.mat

### Experiment directory

Each file in the directories specified in Table 6 corresponds to a given experiment number, the details of which are provided in Table 2.

#### WiFi CSI dataset description

This section describes the structure of the data files residing in the wificsi1 (NUC1) and wificsi2 (NUC2) directories. The files are in .mat format and each row in the file corresponds to a received CSI packet. The columns in the dataset have the following headers:

- timestamp: UTC+01 00 timestamp in milliseconds when the CSI packet was captured by the receiver.
- activity: current activity being performed. The activity is specified as a string of characters with no spacing e.g., “background”, “walk”, “sit”, “stand”, “liedown”, “standfromlie”, “bodyrotate”. These correspond to the activity numbers 1, 2, 3, 4, 5, 6 and 7 in the “Details” column in Table 2, respectively. The activity label “noactivity” refers to the case where the person was not performing any activity, that is, his/her body was at rest, for example between activities such as “sitting” and “standing” or “lying down on floor” and “standing up from the floor”. For exp035–exp054, the activity is specified as “Loc1”, “Loc2”, ⋯, “Loc9” (device-free static target localization), “path1”, “path2”, ⋯, “path5” (device-free dynamic target localization), “loc1”, “loc2”, ⋯, “loc6” (device-to-device localization). For these localization experiments, each file also contains a column with header notes which gives more details on the position of the transmitter (tx) and receiver (rx) and the target (if applicable).
- exp_no: experiment number which is specified as “exp_001”, “exp_002”, etc. See Table 2 for more details.
- person_id: person ID specified as “One”, “Two”, “Three”, etc.
- room_no: room ID specified as “1” (left room in Fig. 1) or “2” (right room in Fig. 1).
- tag4422_x, tag4422_y, tag89b3_x, tag89b3_y: refer to the ground truth position of the person in the monitoring area in terms of 2D *x*- and *y*- coordinates. Note that for all experiments, except exp001, exp019, exp028, and exp034–exp055, the person was wearing two UWB tags on either arms, bearing IDs 4422 and 89b3. The information regarding which tag is worn on which arm is given in the columns with headers left_arm_tag_id and right_arm_tag_id. For the crowd counting experiment (exp028), there were a maximum of 6 people and hence 6 UWB tags were used to obtain the ground truth position of each person. Each person wore the tag on his/her left arm. In the WiFi CSI files for exp028, the *x*- and *y*- coordinates of the person are given in the columns tag4422_x, tag4422_y, tag89b3_x, tag89b3_y, tag122c_x, tag122c_y, tag4956_x, tag4956_y, tag1e85_x, tag1e85_y, and tag9118_x, tag9118_y. The person bearing UWB tag ID 4956 was the first to step out of the monitoring area, followed by 9118, 1E85, 4422, 89B3, and finally 122C.
- anchor_node_xy_positions: *x*- and *y*- coordinates of the eleven UWB anchor nodes distributed across the rooms (see red nodes in Fig. 1) for obtaining the ground truth position of the tag/s.
- tx1rx1_sub1, tx1rx1_sub2, ⋯, tx3rx3_sub30 (270 columns): The first corresponds to the raw complex CSI values for transmit antenna 1 (tx1), receive antenna 1 (rx1) and subcarrier 1 (sub1), the second corresponds to transmit antenna 1 (tx1), receive antenna 1 (rx1) and subcarrier 2 (sub2), and so on. The WiFi CSI systems used a 3 × 3 MIMO configuration and since the Intel 5300 NIC extracts CSI data over 30 subcarriers, the total number of complex CSI values per packet is 3 × 3 × 30 = 270.
- tx_x_coord, tx_y_coord, target_x_coord, target_y_coord (specified for exp035–exp054 only): For exp035–exp048, tx_x_coord and tx_y_coord respectively correspond to the *x*- and *y*- coordinates of both the CSI transmitter (NUC3) and CSI receiver (NUC2) since they were placed side by side while the target was standing still at several positions or walking along a short path. The human target was holding a tag and its ground truth *x*- and *y*- coordinates are given by target_x_coord and target_y_coord, respectively. As for exp049–exp054, tx_x_coord and tx_y_coord refer to the *x*- and *y*- coordinates of the CSI transmitter (NUC3), respectively, while target_x_coord and target_y_coord refer to the *x*- and *y*- coordinates of the CSI receiver (NUC2), respectively. No human target was present in this case.

#### UWB dataset description

This section describes the structure of the data files residing in the uwb1 (UWB system 1- yellow nodes) and uwb2 (UWB system 2- blue nodes) directories. The files are in .csv format and each row in the file corresponds to a received UWB packet. The UWB dataset files have the following fields similar to the WiFi CSI datasets: timestamp, activity, exp_no, person_id, room_no, tag4422_x, tag4422_y, tag89b3_x, tag89b3_y, tag122c_x, tag122c_y, tag4956_x, tag4956_y, tag1e85_x, tag1e85_y, tag9118_x, tag9118_y, left_arm_tag_id, right_arm_tag_id, and anchor_node_xy_positions. The additional column headers or those that are different from the WiFi CSI dataset headers are described below:

- fp_pow_dbm: estimate of the first path power level (in dBm) of the UWB signal between a pair of nodes. The formula for computing this value is given in^44^.
- rx_pow_dbm: estimate of the receive power level (in dBm) of the UWB signal between a pair of nodes. The formula for computing this value is given in^44^.

According to the manufacturer, the above two estimated parameters can be used to infer whether the received signal is Line-of-Sight (LoS) or Non-Line-of-Sight (NLoS). It is stated that, as a rule of thumb, if the difference of the two parameters, i.e., rx_pow_dbm - fp_pow_dbm is less than 6 dB, the signal is most likely to be LoS, whilst if the difference is greater than 10 dB, the signal is likely to be NLoS^44^.

- tx_id: index of the transmitting node. For UWB system 1 (yellow nodes), the transmitting node IDs are 0, 1, 2 or 3. For UWB system 2 (blue nodes), the transmitting node IDs are 0, 1, 2, 3 or 4 (see Fig. 1).
- rx_id: index of the receiving node. For UWB system 1 (yellow nodes), the receiving node IDs are 0, 1, 2 or 3. For UWB system 2 (blue nodes), the receiving node IDs are 1, 2, 3 or 4.
- tx_x_coord,tx_y_coord: *x*- and *y*- coordinates of the transmitting node, respectively.
- rx_x_coord,rx_y_coord: *x*- and *y*- coordinates of the receiving node, respectively.
- tx_rx_dist_meters: separation distance between the pair of transmitting and receiving nodes in meters.
- fp_index: accumulator first path index as reported by the Leading Edge Detection (LDE) algorithm of the DW1000 UWB chipset in register 0x15 (in FP_INDEX field). It is a sub-nanosecond quantity, consisting of an integer part and a fractional part.
- fp_amp1: first path amplitude (point 3) value reported in the FP_AMPL1 field of register 0x15 of the DW1000 UWB chipset.
- fp_amp2: first path amplitude (point 2) value reported in the FP_AMPL2 field of register 0x12 of the DW1000 UWB chipset.
- fp_amp3: first path amplitude (point 1) value reported in the FP_AMPL3 field of register 0x12 of the DW1000 UWB chipset.

Basically, fp_amp1, fp_amp2 and fp_amp3 are the magnitudes of the accumulator tap at the indices 3, 2 and 1, respectively, beyond the integer part of FP_INDEX reported in register 0x15 of the DW1000 UWB chipset^44^. That is, fp_amp1 = amplitude at ceiling(FP_INDEX) + 3, fp_amp2 = amplitude at ceiling(FP_INDEX) + 2 and fp_amp3 = amplitude at ceiling(FP_INDEX) + 1.

- max_growth_cir: Channel Impulse Response (CIR) power value reported in the CIR_PWR field of register 0x12 of the DW1000 UWB chipset. This value is the sum of the squares of the magnitudes of the accumulator from the estimated highest power portion of the channel, which is related to the receive signal power^44^.
- rx_pream_count: Preamble Accumulation Count (PAC) value reported in the RXPACC field of register 0x10 of the DW1000 UWB chipset. RXPACC reports the number of accumulated preamble symbols. The DW1000 chip estimates the CIR by correlating a known preamble sequence with the received signal and accumulating the result over a time period. The number of preambles used for the CIR estimation is dependent on the quality of the received signal.
- max_noise: LDE maximum value of noise.
- std_noise: standard deviation of noise.
- cir1, cir2, ⋯: These correspond to the 35 and 50 raw complex CIR samples for UWB systems 1 and 2, respectively.

#### PWR dataset description

This section describes the structure of the data files residing in the pwr directory. The files are in .mat format and each row in the files corresponds to a PWR measurement from each of the three receivers (surveillance channels) at a given point in time.

**Fig. 5:**
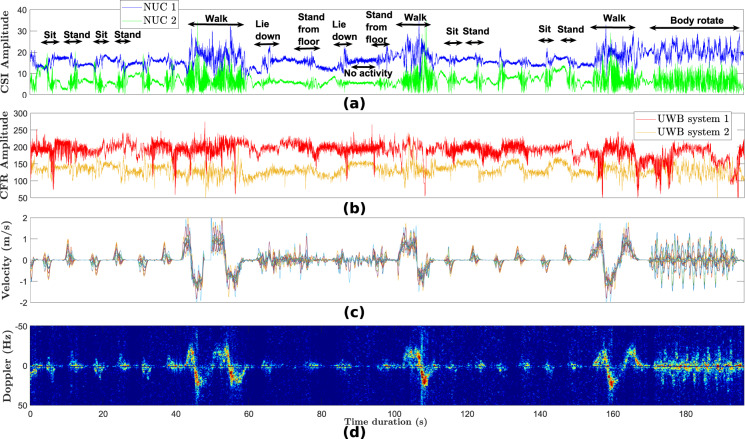
Signal analysis: (**a**) WiFi CSI data (considering transmit antenna 1, receive antenna 1 and subcarrier 10); (**b**) UWB CFR data (considering the 10^th^ CFR sample between node ‘0’ and node ‘3’ for UWB system 1 and nodes ‘1’ and ‘2’ for UWB system 2); (**c**) Velocity information extracted from Kinect sensor data and (**d**) PWR Doppler spectrogram extracted from surveillance channel ‘rx2’. Only a 196-second portion of exp018 is considered for the four synchronized modalities in this illustration.
- PWR_ch2: Doppler spectrogram measured from surveillance channel “rx3”.
- PWR_ch3: Doppler spectrogram measured from surveillance channel “rx4”.

- exp_no: experiment number which is specified as “exp_002”, “exp_003”, etc. See Table 2 for more details. Note that the PWR system does not need background scan. Hence, background data for “exp_001” and “exp_019” were omitted for the PWR system.
- timestamp: UTC+01 00 timestamp in milliseconds when the Doppler spectrograms were recorded.
- activity: ground truth activity labels. The activity is specified as a string of characters with no spacing e.g., “walk”, “sit”, “stand”, “liedown”, “standfromlie”, “bodyrotate”. These correspond to the activity numbers 1, 2, 3, 4, 5, 6 and 7 in the “Details” column in Table 2, respectively.
- person_id: person ID specified as “One”, “Two”, “Three”, etc.
- room_no: room ID specified as “1” (left room in Fig. 1) or “2” (right room in Fig. 1).
- PWR_ch1: Doppler spectrogram measured from surveillance channel “rx2”, as demonstrated in Fig. 5(d).

#### Kinect dataset description

This section describes the structure of the data files residing in the kinect directory. The files are in .mat format and each row in the files corresponds to three-dimensional skeleton information captured from each of the two Kinects at a given point in time.

- exp_no: experiment number which is specified as “exp_002”, “exp_003”, etc. See Table 2 for more details. Note that the Kinect system does not need background scan. Hence, background data for “exp_001”, “exp_019” and “exp_055” were omitted for the Kinect system.
- timestamp: UTC+01 00 timestamp in milliseconds when the Kinect skeleton data was recorded.
- activity: ground truth activity labels. The activity is specified as a string of characters with no spacing e.g., “walk”, “sit”, “stand”, “liedown”, “standfromlie”, “bodyrotate”. These correspond to the activity numbers 1, 2, 3, 4, 5, 6 and 7 in the “Details” column in Table 2, respectively.
- person_id: person ID specified as “One”, “Two”, “Three”, etc.
- room_no: room ID specified as “1” (left room in Fig. 1) or “2” (right room in Fig. 1).
- Kinect1: velocity-time profile derived from Kinect skeleton data over a period of time as demonstrated in Fig. 5(c). It perfectly captures human motion characteristics and is qualitatively similar to the envelope of the human-micro-Doppler signatures presented in Fig. 5(d).

## Technical Validation

### WiFi CSI

Figure 5(a) shows a 196-second portion of the received WiFi CSI signals on NUC1 and NUC2 forexp018. CSI values for transmit antenna 1, receive antenna 1 and subcarrier 10 have been considered here. The injection packet rate of WiFi CSI was set a 1600 Hz. For illustration purposes only, the CSI data has been filtered using a 1D wavelet denoising technique and the corresponding results are shown in Fig. 5(a). It can be observed that the CSI measurements in the time domain capture variations in the wireless signal due to the latter’s interaction with surrounding objects and human bodies. Therefore, machine or deep learning algorithms can be used to train the observed patterns and automatically extract features from raw signals to predict human activities. The CSI data can be processed and interpreted in different ways (feature extraction), for example spectrograms which are generated by applying Short Time Fourier Transform (STFT) to the CSI amplitude data^8–10^. Note that the two receiving NUCs (NUC1 and NUC2) were arranged differently with respect to the transmitter (NUC3). As shown in Fig. 1, in both rooms NUC1 was facing the transmitter in a 180° configuration while NUC2 was in a bistatic geometry (90°) with respect to the transmitter. By deploying multiple receivers in the monitoring area, it is envisaged that the activity prediction accuracy will be improved using multiview data fusion models^55^.

### UWB

Figure 5(b) shows the UWB signals between node ‘0’ and node ‘3’ for UWB system 1 and nodes ‘1’ and ‘2’ for UWB system 2, considering the same experiment number and time window. The raw CIR data has been converted to Channel Frequency Response (CFR) using the Fast Fourier Transform (FFT) and the signals are plotted for the 10^th^ CFR sample for each system. Note that the terms CFR and CSI can be used interchangeably. Although the sampling rate of the UWB systems is much lower (typically <100 Hz considering bidirectional data which are reciprocal) than the WiFi CSI system, the activities cause variations in the UWB signals and these variations can be fed to machine or deep learning algorithms for activity prediction. The raw CIR can also be used for human activity recognition (HAR) and yield high prediction accuracy, as demonstrated in^10^.

**Fig. 6:**
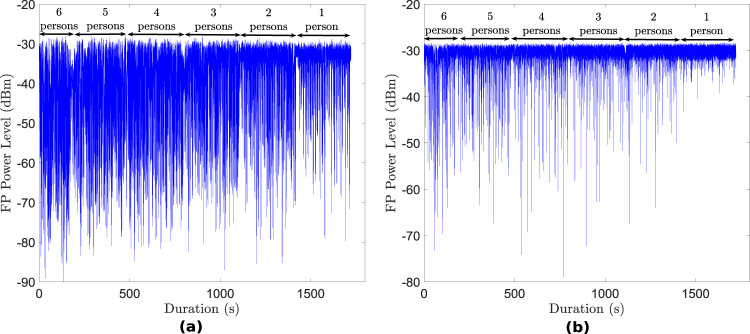
First path power level (dBm) of UWB signal in crowd counting experiment (exp028) between nodes (**a**) ‘0’ and ‘3’ of UWB system 1 (yellow nodes) and (**b**) ‘3’ and ‘4’ of UWB system 2 (blue nodes).

Considering the crowd counting experiment, Fig. 6 shows the first path power level (in dBm) for the two UWB systems between a given pair of nodes in each case. The first path power level (fp_pow_dbm) has been computed using the formula given in the DW1000 manual^44^. As can be observed, the first path power level increases gradually as each person was moving out of the monitoring area. This is an expected behaviour since the LoS signal becomes less and less obstructed. By using the fp_pow_dbm parameter together with other parameters such as overall received UWB signal power level (rx_pow_dbm), UWB CIR data and WiFi CSI data, the number of people in a given environment can be inferred through the use of artificial intelligence algorithms.

**Fig. 7:**
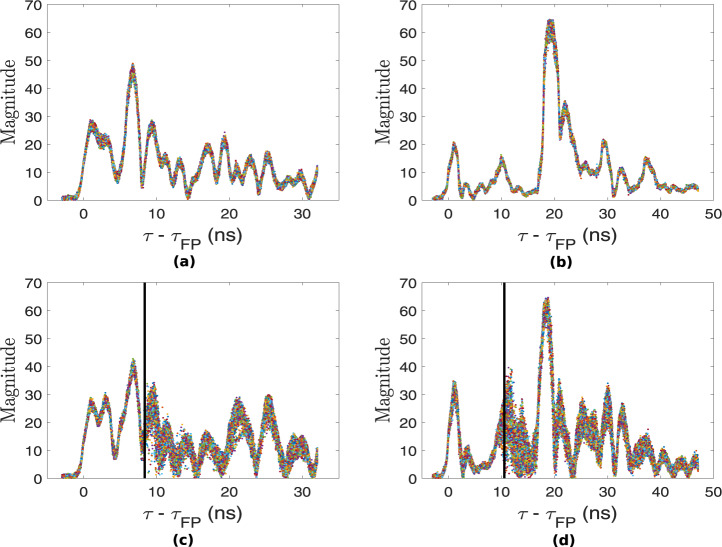
1000 accumulated and aligned CIR measurements in a (**a**) static environment (exp001) recorded between nodes ‘1’ and ‘3’ of UWB system 1 (yellow nodes); (**b**) static environment (exp001) recorded between nodes ‘2’ and ‘3’ of UWB system 2 (blue nodes); (**c**) dynamic environment (exp003) recorded between nodes ‘1’ and ‘3’ of UWB system 1 (yellow nodes) and (**d**) dynamic environment (exp003) recorded between nodes ‘2’ and ‘3’ of UWB system 2 (blue nodes). Note: Bidirectional CIR data are reciprocal. *τ*_FP_ represents the first path (direct) signal time-of-flight between the pair of nodes.

While UWB modules such as Decawave’s EVK1000 and DWM1000 are used for active localization to provide the 2D or 3D coordinates of a target carrying a tag, in this experiment, we deployed fixed UWB nodes programmed with custom software to record CIR data in a multi-static configuration. The idea is to extend the functionality of pulse-based UWB systems from active localization to the ability to sense their environment using the CIR data^23,45,56^. Figure 7(a,b) show 1000 aligned and accumulated CIR measurements recorded between a given pair of UWB modules for each system in a static environment (exp001). As can be observed from Fig. 7(a,b), when the room is empty, the accumulated CIR measurements are stable. However, when a person is performing activities as in exp003, some variations occur in the accumulated CIR, as can be observed in the region starting around *τ-τ*_FP_ ≈ 8 ns and 10 ns in Fig. 7(c,d), respectively. The earliest time at which changes are observed in the CIR is the bi-static delay. Since the transceivers are fixed in the multi-static network and their positions are known, the distance travelled by the direct (first path) signal between pairs of devices can be computed along with its delay *τ*_FP_. The black vertical lines in Fig. 7(c,d) represent the ground truth bi-static delay which is computed from the tags’ coordinates. Note that the target was wearing two tags, one on each arm. Also, exp003 refers to the sitting on chair and standing up from chair activities which were performed at a given location within the monitoring area. Therefore, the reported locations for each tag were averaged over the 1000 accumulated CIR measurements to obtain a single 2D position per tag. Now, the coordinates of the two tags can be used to compute the separation distance between them, which is around 60 cm, corresponding to the approximate diameter of the human (arm to arm distance). Therefore, the midpoint *xy* coordinates are taken as the ground truth position of the target. The ground truth bi-static delay can then be computed by finding the transmitter-target-receiver path length and subtracting the direct signal path length from it, given that the fixed UWB transmitter and receiver positions are known. Assuming that the signal emitted from the transmitter reflects off the target and reaches the receiver without additional scattering, then the bi-static range defines an ellipse on which the target is located^23^. This ellipse has the position of the transmitter and receiver as foci points and the major axis length is equal to the bi-static range. In an ideal scenario, the common intersection point of ellipses from multiple transmitter and receiver pairs indicates the location of the target.

It should be noted that in the multi-static UWB network, each transceiver device runs its own independent RF clock and therefore CIR measurements between pairs of devices may be sampled at different times^23^. The DW1000 chip organizes the CIR buffer in such a way that the reported first path index (FP_INDEX) in each CIR measurement is usually around 750^57^. The chipset also estimates FP_INDEX in each CIR measurement with a resolution of $\frac{1.0016\;{\rm{ns}}}{64}$, such that it is represented as a real number, having integer and fractional parts (see column fp_index in UWB datasets). Now, since each CIR measurement basically has a different FP_INDEX value but the same sampling resolution of 1.0016 ns, the accumulated CIR measurements need to be aligned with their respective estimated FP_INDEX, and the latter can be shifted to be at the beginning of the CIR buffer, as shown in Fig. 7. Furthermore, in order to remove outliers in the accumulated CIR, those CIR measurements where the number of accumulated preamble symbols (see column rx_pream_count in UWB datasets) is less than half of the number of transmitted preamble symbols can be discarded^23^ (preamble length of 128 considered in the experiments).

### PWR

Figure 5(d) illustrates the Doppler spectrogram collected by the PWR system from a given angle (see placement of “rx2”, “rx3” and “rx4” in Fig. 1). As mentioned previously, the PWR system uses the CSI transmitter (NUC3) as the signal source with an injection packet rate of 1600 Hz. The coherent processing interval was set at 1 second, and sampling rate was set at 20 MHz for each channel. The signatures in the Doppler spectrogram demonstrate the relative velocity between the human and receiver antenna. The bi-static velocity is maximum in a monostatic layout (0°), and minimum in a forward scatter layout (180°). We can see large differences between the signatures from the “sit/stand” and “walk” activities in terms of Doppler frequency shifts. This is because the velocity of the walking activity is much higher than other activities. The activities “lie down” and “stand from floor” have opposite Doppler signatures due to the opposing directions the human body undertakes when performing these activities. Such signatures can potentially be used for various machine learning applications such as activity recognition, people counting, localization, etc.

### Kinect

Figure 5(c) illustrates the velocity-time profile of a human undergoing a set of activities. The motion profile is generated using the time-varying three-dimensional position information of different joints on the human body, such as the torso, arms, legs, and the head, at a frame rate of 10 Hz. To mimic accurate radar reflections from the target, we assume the radar scattering centers lie approximately at the center of the bones joining these joints. Thus, the signatures in the velocity-time plot demonstrate the relative velocity between human scattering centers and the Kinect position.

Figure 5(c,d) compare the velocity-time profile (generated using motion capture data) and the measured spectrograms, respectively, for a human undergoing a series of motions. The envelope of the velocity-time profile is visually very similar to the measured spectrogram indicating how well both systems capture the motion characteristics. For example, as the human sits down, we observe negative Doppler due to the bulk body motion. The positive micro-Doppler arises due to arm motion and legs moving slightly in the sensor direction while sitting down. After a 5-second delay, the human subject stands up from the chair, resulting in primarily positive Dopplers. Similarly, the latter part of the spectrogram presents signatures corresponding to a human transitioning from first walking to lying down and then standing up from the ground to rotate his body while standing at a fixed position.

**Fig. 8:**
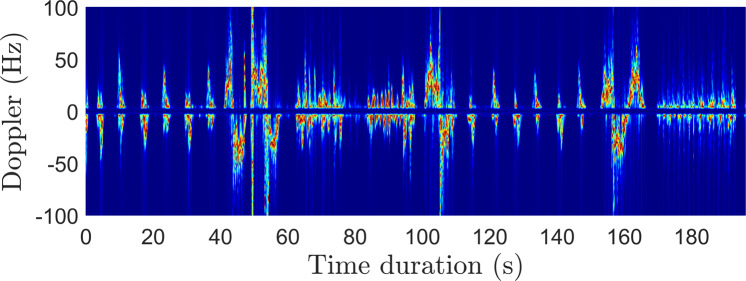
Micro-Doppler signature produced by *SimHumalator*^52^ using real motion capture data from the Kinect system. In this illustration, a 196-second portion of the Kinect data in exp018 is considered.

In most realistic scenarios, the human motions might not be restricted to a single aspect angle with respect to the radar. In such scenarios, the spectrograms might differ significantly. It could be due to the shadowing of some part of the human body if captured at different angles. Therefore, we can leverage the animation data captured by the Kinect to feed as input to our human radar simulator, *SimHumalator* and synthesize radar returns as a function of different target parameters (varying aspect angles) and different sensor parameters (varying bi-static angles and operational parameters such as waveform design and antenna characteristics). Figure 8 shows an example of the micro-Doppler signature produced by *SimHumalator* when real motion capture data from the Kinect system is used as input to the software. It can be observed that the generated Doppler signature in Fig. 8 is very similar to the real PWR spectrogram data in Fig. 5(d). Therefore, such signatures can potentially augment otherwise limited real radar databases for various machine learning applications such as activity recognition, people counting, and identification.

### Human activity recognition (HAR) classification performance

**Fig. 9:**
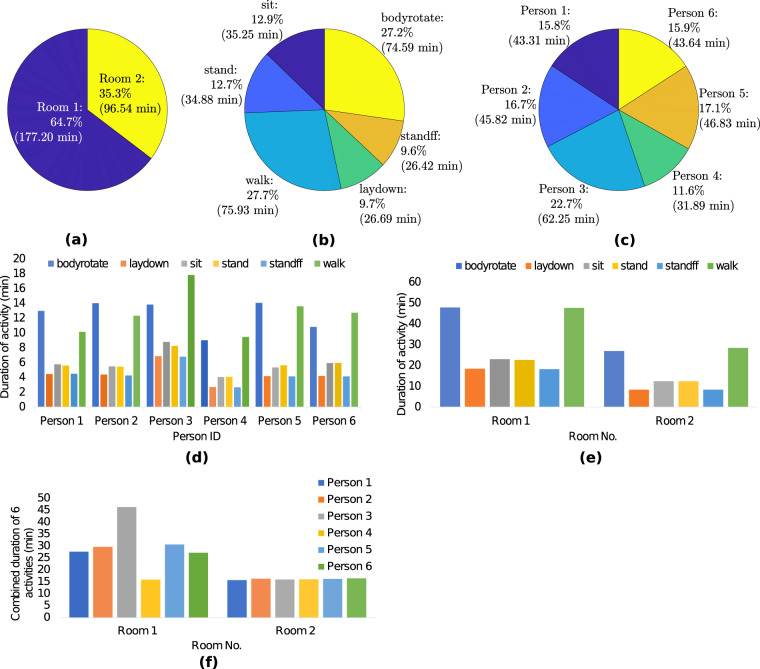
Distribution of the 6 activities performed by the 6 participants in the 2 rooms: (**a**) overall room distribution; (**b**) overall activity distribution; (**c**) overall participant distribution; (**d**) distribution of activities per participant; (**e**) distribution of activities in each room and (**f**) distribution of participants in each room.

In this section, we apply a mainstream algorithm such as Convolutional Neural Network (CNN) on the proposed dataset to verify its potential usefulness for the activity recognition task and as such provide a baseline. Essentially, we use the ResNet-18 CNN on the data collected from the WiFi CSI, Kinect and PWR modalities to perform classification on the six human activities, namely, sitting down on a chair (“sit”), standing from the chair (“stand”), laying down on the floor (“laydown”), standing from the floor (“standff”), upper body rotation (“bodyrotate), and walking (“walk”). Figure 9 shows various statistics regarding the distribution of the six activities performed by the six participants in the two rooms. It can be observed from Fig. 9(a) that more time was spent doing the six activities in Room 1. Figure 9(b) shows that the walking and body rotating activities were performed more frequently. It can also be observed from Fig. 9(c) that the participants shared a fair amount of time performing all six activities. Finally, it can be deduced from Fig. 9(d–f) that the six participants performed all six activities in both rooms, and in Room 2 each person performed the activities in approximately the same amount of time.

**Fig. 10:**
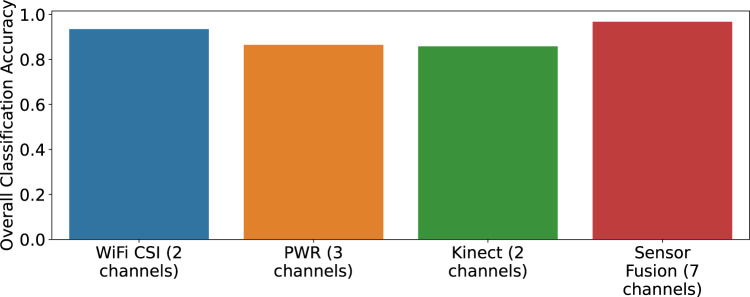
Human Activity Recognition (HAR) classification accuracy.

**Fig. 11:**
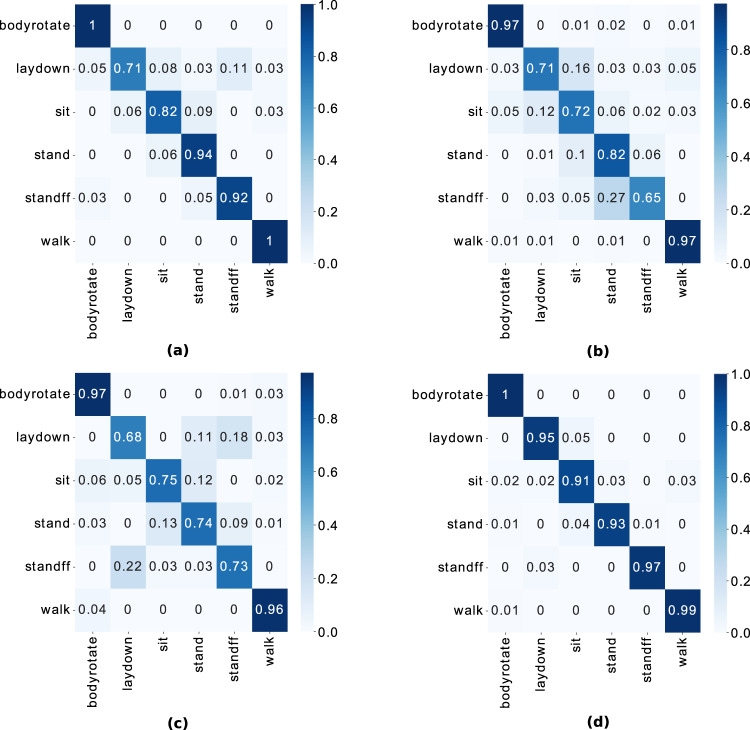
Confusion matrices depicting the HAR performance: (**a**) WiFi CSI (2 channels); (**b**) PWR (3 channels); (**c**) Kinect (2 channels) and (**d**) sensor fusion (7 channels).

We convert the raw data from these three modalities into image-like format known as spectrograms, as illustrated in Fig. 5(d) and Fig. 8. The interested reader is kindly referred to the signal processing pipeline described in our previous works^8,9,48^ for converting WiFi CSI data into spectrograms. It should be noted that several feature extraction methods and machine/deep algorithms can be used for performing HAR classification using WiFi CSI data^58–61^. For each modality, the synchronized spectrogram data are segmented into 4 seconds duration windows using the activity labels and then reshaped into a dimension of *N* × 1 × 224 × 224, where *N* represents the number of samples. For each modality/system, we consider each receiver data as a one-channel image. For instance, the WiFi CSI system consists of two receivers (NUC1 and NUC2), therefore the spectrogram data from the two receivers are concatenated along the channel dimension, resulting into an image dimension of *N* × 2 × 224 × 224. The same feature fusion technique is used for the PWR system (3 surveillance channels) and Kinect system (2 receivers) independently. Thus, the spectrograms serve as a multi-channel image input to the ResNet-18 CNN. We train the model in a supervised fashion for each modality separately. We also perform a simple sensor fusion technique where we concatenate all modalities data (for all receivers) along the channel dimension, resulting into a 7-channel CNN model. In each case, the ResNet-18 model is trained over 70 epochs using the Adam optimizer with a learning rate of 1e-5, weight decay of 0.001, and *β*_1_ = 0.95 and *β*_2_ = 0.999. A batch size of 128 and cross-entropy loss function are also considered during training. 80% of the spectrogram data are used for training and the remaining 20% for testing. When each modality is trained independently, the same random seed is used for the train/test split to ensure fair comparison. The HAR classification accuracy is reported in Fig. 10 for the case when the three modalities are trained separately, as well as for the sensor fusion method. The corresponding confusion matrices are also shown in Fig. 11. Considering the separately trained modalities, it can be observed from Fig. 10 that the WiFi CSI modality achieves the highest accuracy (93.5%) while the PWR and Kinect modalities achieve comparable performance with accuracy values of 86.5% and 85.8%, respectively. On the other hand, the fusion of the three modalities data improved the overall performance, resulting in an accuracy as high as 96.7%. The benefit of sensor fusion is also reflected in Fig. 11(d) where it can be observed that the performance across the six different activities are greatly improved. These results serve as a baseline and the users of this dataset are encouraged to explore different feature extraction methods and algorithms in order to yield improved performance. Furthermore, various multimodal sensor fusion methods (decision-level fusion or feature-level fusion)^62–65^ can be investigated on this dataset.

For the sake of brevity, we omitted the UWB modality in this evaluation, although the UWB CIR data can as well be used effectively for activity recognition, as we have shown in our previous work^10^. The UWB CIR data reported by the Decawave’s chipsets is very attractive for passive (non-cooperative) target localisation, as we have demonstrated in our previous work^56^, where we use a series of well-defined signal processing steps to passively track a person with good localization accuracy using only the reported CIR data (sampling resolution of the complex CIR samples is ≈ 1 ns). We can leverage the variance in the CIR data (refer to Fig. 7) to find the time-of-flight (ToF) of the signal (caused by moving target) between each transmitter-receiver link and combine them, for example using Taylor Series or intersection of ellipses method, along with Kalman tracking or particle filtering to find the 2D coordinates of the moving target^23,56^.

## Usage Notes

The different directories available in our curated dataset^39^ are specified in Table 6. Furthermore, the interested reader is encouraged to navigate to the codes directory where example scripts on how to load and analyze a specific modality data are included. These are described in the following section .

**Fig. 12:**
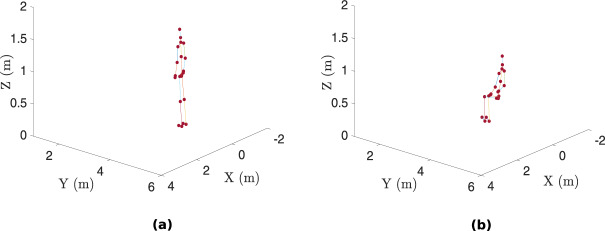
Example of stick representations of the Kinect motion capture data: (**a**) person standing and (**b**) person sitting.

## Data Availability

Some Matlab and Python scripts have been made available in the codes directory for the users to replicate some of the figures in this Data Descriptor: • plot_wificsi.m: This script is used to load the complex WiFi CSI data recorded by each NUC device and visualize the amplitude variations over time, as illustrated in Fig. 5(a). The user can specify the start and stop timestamps and visualize the CSI stream in that time segment (for a given transmit antenna, receive antenna, and subcarrier index). Furthermore, for comparison purposes, the generated plots consist of the raw (unfiltered) CSI data and those which have been denoised using Discrete Wavelet Transform (DWT). • plot_uwb.m: This script is used to load the complex CIR data recorded by each passive UWB system, convert it into CFR using FFT and visualize the amplitude variations over a given time segment (between a given pair of UWB nodes), as illustrated in Fig. 5(b). Furthermore, this script allows the users to plot the accumulated and aligned CIR measurements, as shown in Fig. 7. • plot_uwb_fppow_crowdcount.m: This script is used to load the UWB data for the crowd counting experiment (exp028) and plot the first path power level (in dBm) over time for each UWB system (between a given pair of UWB nodes), as illustrated in Fig. 6. • plot_PWR_demonstration.m and plot_pwr_spectrogram.py: These scripts allow the users to visualize the PWR spectrograms from the three surveillance channels: “rx2” (as illustrated in Fig. 5(d)), “rx3” and “rx4”, as a function of time and Doppler. • plot_kinect_data.m: This script allows the user to plot the motion capture data (as a function of velocity versus time) from one of the two Kinect systems, as illustrated in Fig. 5(c). Furthermore, the users can visualize the stick (skeletal) representation of the kinect motion capture data as an animation over the specified time segment. For example, two frames of the stick representations when a person is standing and sitting are illustrated in Fig. 12(a,b), respectively. • oddet.py: This python script allows the user to extract only the modalities and features needed rather than having to load the entire files and then stripping out unused features. With this python script, one can select the modality, experiment number and features needed through the command line interface. Additionally if a specific set of features are required, one can also specify all the columns needed through YAML configurations which will allow the user to curate the dataset to the format that more closely suits the usage. This python script is available at the following GitHub repository: https://github.com/RogetK/ODDET.
